# Pancreatic Exocrine Tissue Architecture and Integrity are Maintained by E-cadherin During Postnatal Development

**DOI:** 10.1038/s41598-018-31603-2

**Published:** 2018-09-07

**Authors:** Jeffrey D. Serrill, Maike Sander, Hung Ping Shih

**Affiliations:** 1 Department of Translational Research and Cellular Therapeutics, Diabetes and Metabolic Research Institute, Beckman Research Institute, City of Hope, Duarte, CA 91010 USA; 2 Departments of Pediatrics and Cellular and Molecular Medicine, Pediatric Diabetes Research Center, Sanford Consortium for Regenerative Medicine, University of California, San Diego, CA 92093 USA

## Abstract

Cadherin-mediated cell-cell adhesion plays an important role in organ development and changes in cadherin expression are often linked to morphogenetic and pathogenic events. Cadherins interact with other intracellular components to form adherens junctions (AJs) and provide mechanical attachments between adjacent cells. E-cadherin (Cdh1) represents an integral component of these intercellular junctions. To elucidate the function of E-cadherin in the developing pancreas, we generated and studied pancreas-specific *Cdh1*-knockout (*Cdh1*^*ΔPan/ΔPan*^) mice. *Cdh1*^*ΔPan/ΔPan*^ mice exhibit normal body size at birth, but fail to gain weight and become hypoglycemic soon afterward. We found that E-cadherin is not required for the establishment of apical-basal polarity or pancreatic exocrine cell identity at birth. However, four days after birth, the pancreata of *Cdh1*^*ΔPan/ΔPan*^ mutants display progressive deterioration of exocrine architecture and dysregulation of Wnt and YAP signaling. At this time point, the acinar cells of *Cdh1*^*ΔPan/ΔPan*^ mutants begin to exhibit ductal phenotypes, suggesting acinar-to-ductal metaplasia (ADM) in the E-cadherin-deficient pancreas. Our findings demonstrate that E-cadherin plays an integral role in the maintenance of exocrine architecture and regulation of homeostatic signaling. The present study provides insights into the involvement of cadherin-mediated cell-cell adhesion in pathogenic conditions such as pancreatitis or pancreatic cancer.

## Introduction

During the coordinated processes of development and morphogenesis, epithelial cells engage in dynamic rearrangements of their intercellular connections with neighboring cells. These rearrangements facilitate the ongoing changes in movement and cell shape that must occur during epithelial remodeling and tissue establishment. Conversely, this structural remodeling tends to be restricted in mature epithelial tissues, where static tissue maintenance plays a more central role than dynamic growth and reorganization^1–5^. In the developing pancreas, a transition between these distinct structural states occurs as the organ develops from a primordial embryonic bud into a highly branched, functionally specified epithelial organ^6^. Throughout this process, dynamic changes in the adhesive properties of cells coincide with the differentiation, expansion and maintenance of the distinct lineages of the pancreas, including the endocrine, acinar and ductal cells. Central to the process of epithelial adhesion is the adherens junction (AJ), where the interaction of E-cadherin (Cdh1) with cytoplasmic proteins (e.g. α-catenin, β-catenin, δ-catenin) helps to regulate cytoskeletal dynamics, intracellular signaling and gene transcription^7–9^. In individual cells of the pancreas, the extracellular domain of E-cadherin homophilically interacts with complementary cadherin domains of neighboring cells, linking their actin cytoskeletons into functional epithelial tissues. In this regard, cadherin-mediated cell-cell adhesion plays an integral role in the processes of organ development and maintenance.

Previous studies have demonstrated that alterations in cadherin expression are often linked to morphogenic or pathogenic events^10–12^. Through their roles as junctional components, E-cadherin and the AJ are known to play roles in regulating complex intracellular signaling dynamics in epithelial tissues, including homeostatic signaling crosstalk between the Wnt and Hippo/YAP pathways^13,14^. In the pancreas, these pathways must be highly coordinated at the AJ, and a number of studies have systematically addressed the requirement of normal Wnt and YAP pathway expression during development^15,16^. Despite these characterizations, a number of models have focused on the deletion of discrete components of the AJ complex, revealing that, depending on the specific component affected, the subsequent effects to pancreas development and maintenance vary. For instance, deletion of the AJ component β-catenin has been shown to prevent the normal differentiation of the exocrine lineage of the pancreas without affecting the endocrine compartment^16,17^. Similarly, deletion of the β-catenin binding partner α-catenin results in improper endocrine differentiation^18^. In contrast to both of these situations, deletion of δ-catenin (p120-catenin) appears to have a minimal effect on the process of pancreas lineage differentiation, and instead results in widespread dysregulation of pancreas architecture^19^. Through these studies, it has become clear that dysregulation of various AJ components can lead to a wide range of phenotypic changes to normal pancreas development and maintenance. Thus, the roles which individual components of the AJ play in maintaining epithelial homeostasis and preventing pathogenic growth signaling are worthy of further evaluation.

Despite these pioneer studies deciphering the involvement of AJ components in regulating pancreas development, the role of E-cadherin in the development and maintenance of the pancreas remains elusive. To address this knowledge gap, the present study investigated the role of E-cadherin in the development and maintenance of the pancreas. In the present study, we generated a pancreas-specific *E-cadherin* knockout line (*Pdx1Cre; Cdh1*^*flox/flox*^, hereafter: *Cdh1*^*ΔPan/ΔPan*^) and assessed the resulting phenotypic characteristics of pancreas tissue during embryonic and postnatal development. We show that E-cadherin is not required for the initial establishment of the various lineages of the mammalian pancreas, as acinar, ductal, and endocrine lineages all developed through postnatal day 0. By postnatal day 4 however, a progressive deterioration of exocrine architecture became apparent, manifesting in significant reductions to both body weight and blood glucose levels, as well as postnatal lethality which can likely be attributed to pancreatic insufficiency. Through this time period, cells of the *Cdh1*^*ΔPan/ΔPan*^ pancreas were unable to form intercellular adherens junctions (AJs), confirming the requirement of E-cadherin for proper formation of the AJ structure. Furthermore, upregulation of YAP and Wnt signaling pathways were also observed. Finally, we observed evidence of acinar-to-ductal metaplasia (ADM), a known precursor to the progression of pancreatic ductal adenocarcinoma (PDAC). Collectively, the present study provides evidence that E-cadherin, aside from playing a prominent role in intercellular adhesion, also exerts a significant stabilizing effect on pancreas organ homeostasis, and that its inactivation may promote pathogenesis in the pancreas.

## Results

### Pancreatic E-cadherin is required for postnatal growth but not endocrine differentiation

To assess the role which E-cadherin plays during the process of pancreas development, *Cdh1*^*ΔPan/ΔPan*^ mice were generated in which E-cadherin was deleted in *Pdx1-*expressing pancreatic progenitors. In *Cdh1*^*ΔPan/ΔPan*^ mice, E-cadherin expression was almost entirely absent throughout the pancreas in comparison to control littermates by E11.5 (Supp. Fig. 1A,B). Despite this reduction, *Cdh1*^*ΔPan/ΔPan*^ pancreata exhibited all pancreatic cell lineages at E15.5 (Supp. Fig. 1C–F), suggesting that E-cadherin is not required for pancreatic cell differentiation during embryogenesis.

**Figure 1:**
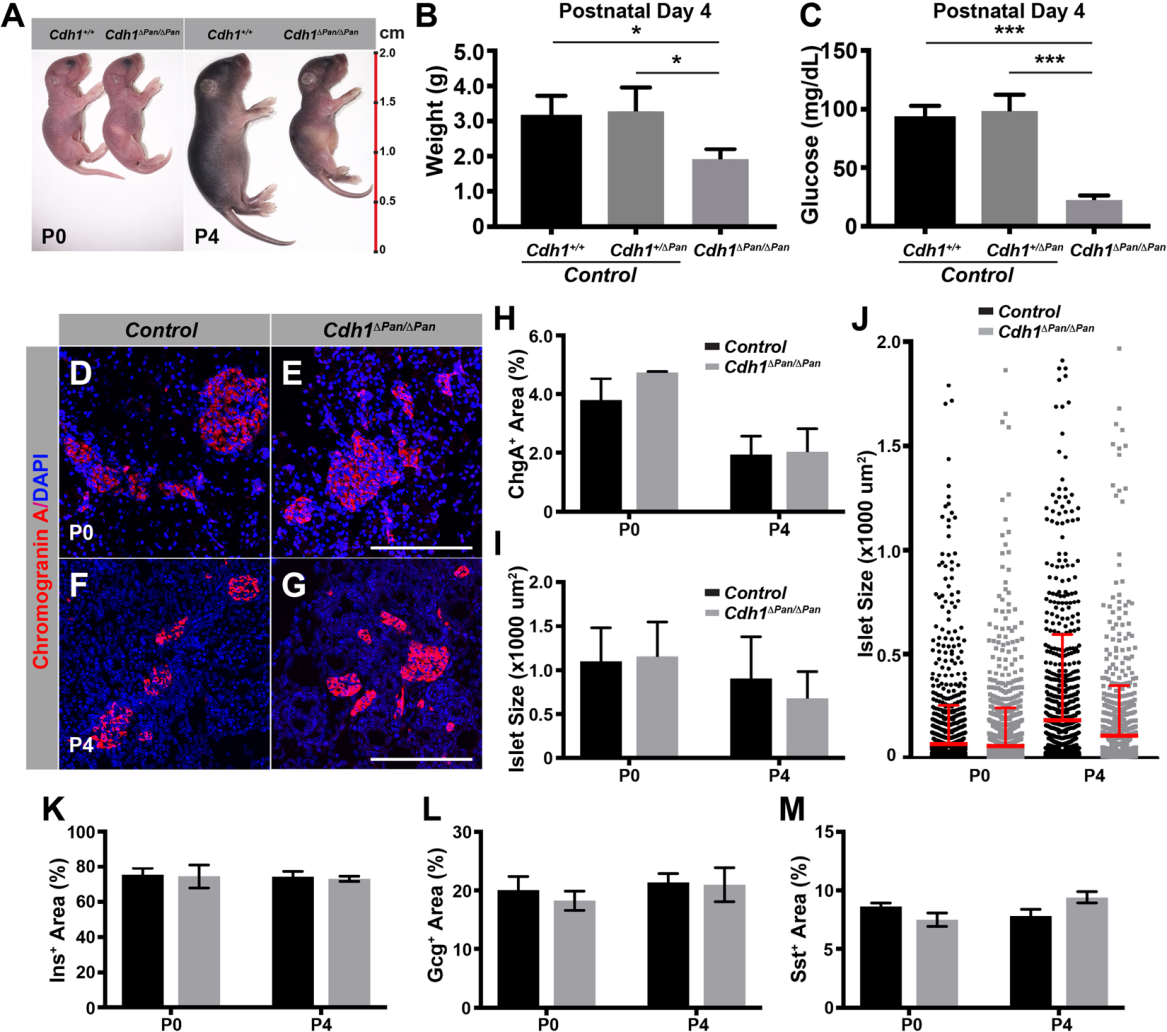
E-cadherin is required for postnatal pancreas growth, but dispensable for the formation of pancreatic islets. (**A**) Gross appearance of control and *Cdh1*^*ΔPan/ΔPan*^ mice at P0 and P4. (**B**) Body weight and (**C**) blood glucose measurements of animals at P4. Immunofluorescence analysis of the endocrine marker chromogranin A (red) in the P0 (**D**,**E**) and P4 (**F**,**G**) pancreas. Nuclear staining (DAPI) shown in blue. White scale bars = 100 μm. Quantification of (**H**) endocrine content and (**I**) islet size in the P0 and P4 pancreas. (**J**) Islet sizes from representative pancreas organs at P0 and P4. Red line and error bar = mean and upper limit of SD. Quantification of (**K**) Insulin, (**L**) Glucagon, and (**M**) Somatostatin relative area in comparison to total Chromogranin A area in the P0 and P4 pancreas. Histograms represent mean ± SEM of at least three independent determinations. For 2-tailed t-tests, *p < 0.05, **p < 0.01, ***p < 0.001 compared to control.

At birth, *Cdh1*^*ΔPan/ΔPan*^ mice were indistinguishable from control littermates in regard to body size. Over the course of several days however, *Cdh1*^*ΔPan/ΔPan*^ mice remained smaller than control littermates and appeared to be malnourished (Fig. 1A,B); these mice died at approximately P4. In addition to a failure to gain weight, *Cdh1*^*ΔPan/ΔPan*^ mice also became hypoglycemic by P4 (Fig. 1C). To determine whether these observations were attributable to improper development of pancreatic endocrine cells, we analyzed the endocrine compartment at both P0 and P4. While the size of P4 *Cdh1*^*ΔPan/ΔPan*^ pancreata was smaller than that of control littermates (Supp. Fig. 1G), the proportion of endocrine tissue relative to pancreas tissue (Fig. 1D–H)), as well as the size of individual islets (Fig. 1I,J) were unaltered. The endocrine cell subtypes were present in *Cdh1*^*ΔPan/ΔPan*^ islets (Supp. Fig. 1H–K), and the proportion of insulin-, glucagon-, and somatostatin-expressing area relative to total chromogranin A-expressing area was unchanged between *Cdh1*^*ΔPan/ΔPan*^ and control islets (Fig. 1K–M). Thus, while elimination of *Cdh1* in pancreatic progenitors results in improper blood glucose regulation, our data suggests that this phenotype is unlikely due to improper endocrine formation or composition. These results suggest that *Cdh1* is dispensable for the formation of pancreatic islets, but is required for the postnatal growth of the pancreas.

### E-cadherin is essential for postnatal exocrine tissue architecture

**Figure 2:**
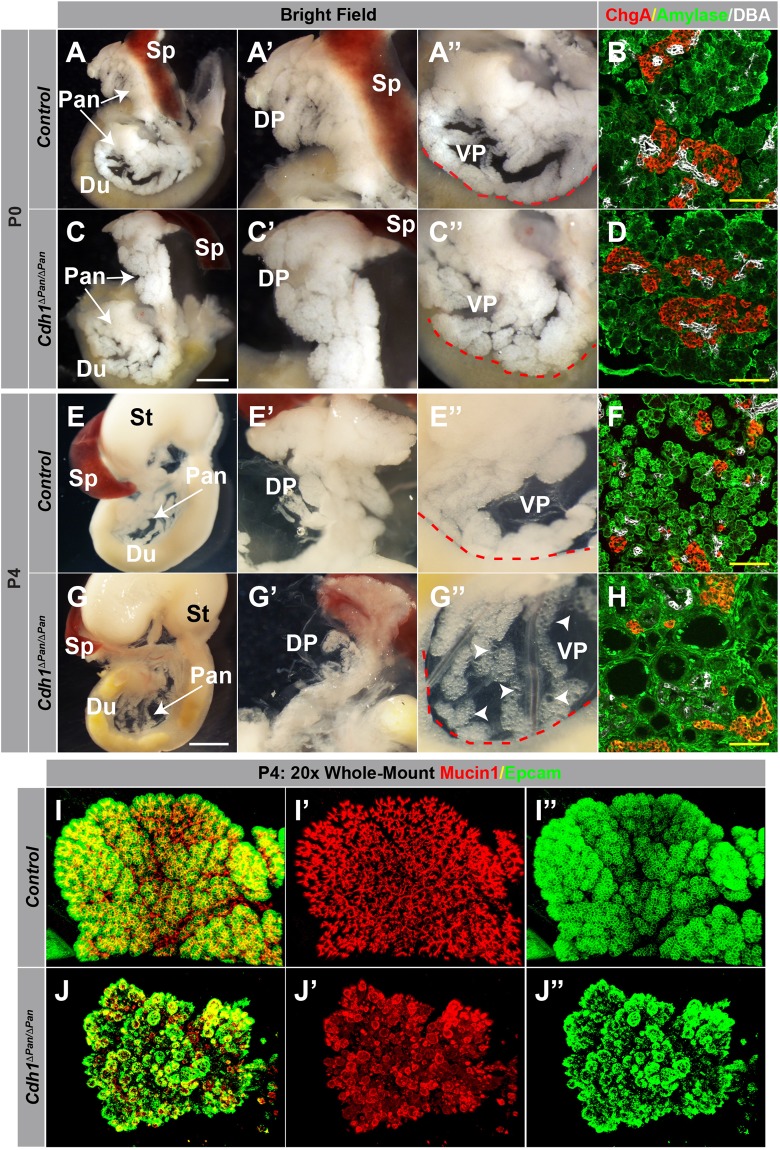
E-cadherin plays an indispensable role in postnatal exocrine tissue architecture. (**A**,**C**,**E**,**G**) Whole-mount images of the pancreas (Pan), spleen (Sp), stomach (St) and duodenum (Du) of control and *Cdh1*^*ΔPan/ΔPan*^ animals at P0 (**A**,**C**) and P4 (**E**,**G**). Higher magnification views of the dorsal (DP, A’,C’,E’,G’) and ventral (VP, A”,C”,E”,G”) pancreas are shown. White scale bars = 250 μm. Red contour indicates edge of duodenum. White arrowheads indicate cystic exocrine structures. (**B**,**D**,**F**,**H**) Immunofluorescence analysis of acinar (amylase, green), ductal (DBA, white), and endocrine (Chromogranin A, red) compartments of control (**B**,**F**) and *Cdh1*^*ΔPan/ΔPan*^ (**C**,**H**) mice at both P0 and P4. Yellow scale bars = 100 μm. (**I**,**J**) Whole-mount immunofluorescence images showing the distribution of ductal (Muc1, red) and exocrine (Epcam, green) compartments in the control (**I**) or *Cdh1*^*ΔPan/ΔPan*^ (**J**) pancreas at P4.

Functionally, the exocrine compartment of the pancreas is essential for nutrient digestion and absorption. Thus, we reasoned that the hypoglycemic phenotype observed in *Cdh1*^*ΔPan/ΔPan*^ mice could be due to malfunction of pancreatic exocrine tissue. To assess the requirement of E-cadherin in postnatal pancreas architecture, we analyzed the gross morphology of *Cdh1*^*ΔPan/ΔPan*^ pancreata at P0 and P4. At P0, the overall morphology of the pancreas was indistinguishable from that of control littermates (Fig. 2A,C). At P4 however, while surrounding organs (stomach, spleen) appeared normal with regard to size and morphology, both the dorsal and ventral pancreas of *Cdh1*^*ΔPan/ΔPan*^ mice demonstrated widespread cystic architectures (Fig. 2E,G). Importantly, this architectural breakdown did not result from alterations to pancreatic lineage development, as the expression of endocrine, acinar, and ductal markers were present in *Cdh1*^*ΔPan/ΔPan*^ pancreata at both P0 and P4 (Fig. 2B,D,F,H, Supp. Fig. 2A–D). To determine whether the architecture of a specific exocrine lineage was disrupted in neonatal *Cdh1*^*ΔPan/ΔPan*^ mice, we analyzed the architecture of exocrine and ductal tissues by whole-mount immunofluorescence analysis. In control pancreata, Epcam^+^ acinus units were clustered at the ends of Mucin1^+^ branched pancreatic ducts (Fig. 2I, Supp. Fig. 2G). While the architecture pancreatic exocrine tissues in P0 *Cdh1*^*ΔPan/ΔPan*^ mice is similar to control littermates (Supp. Fig. 2H), the P4 *Cdh1*^*ΔPan/ΔPan*^ pancreata exhibited an extensive breakdown of exocrine architecture throughout both the acinar and ductal compartments; (Fig. 2J). Thus, E-cadherin plays an essential role in the maintenance of postnatal pancreas architecture but is not required for the development of pancreatic cell lineages.

### Impairment of cell-cell contacts accompanies the progressive dysregulation of postnatal exocrine architecture

**Figure 3:**
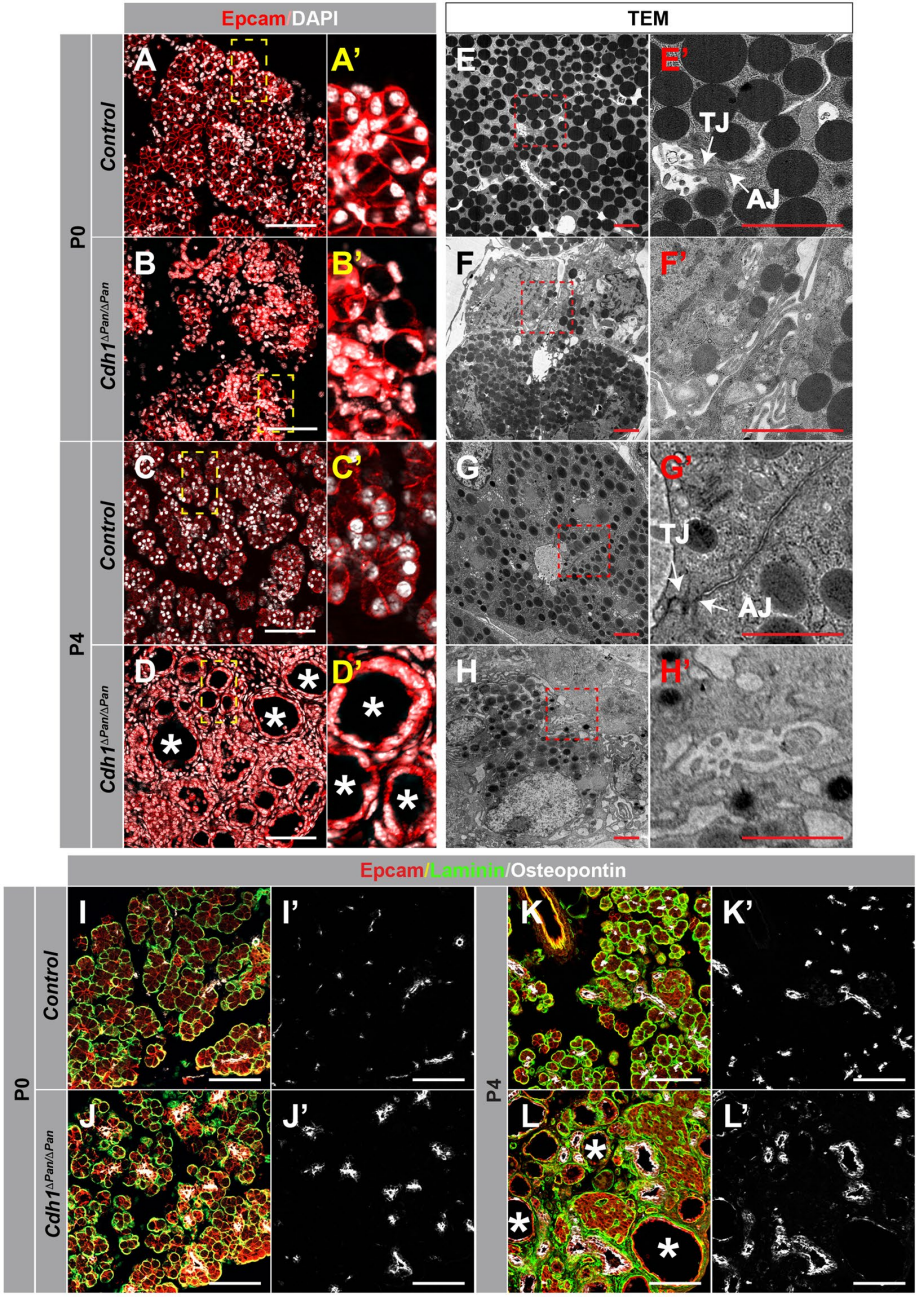
Impairment of adherens junctions and disrupted exocrine tissue integrity in *Cdh1*^*ΔPan/ΔPan*^ pancreata. (**A**–**D**) Immunofluorescence analysis of Epcam (red) and DAPI (white) in control (**A**,**C**) and *Cdh1*^*ΔPan/ΔPan*^ (**B**,**D**) pancreata at P0 and P4. Higher magnification views of yellow inset boxes are shown to the right in (A’,B’,C’,D’). (**E**–**H**) Transmission electron micrographs from control (**E**,**G**) and *Cdh1*^*ΔPan/ΔPan*^ (**F**,**H**) pancreata at P0 and P4. Higher magnification views of red inset boxes are shown to the right in (E’–H’). White arrows point to tight junctions (TJs) and adherens junctions (AJs) as indicated. (**I**–**L**) Immunofluorescence analysis of Epcam (red), Laminin (green), and Osteopontin (white) in the pancreas of control (I,K) or *Cdh1*^*ΔPan/ΔPan*^ (**J**,**L**) mice at P0 and P4. White asterisks indicate dilated/cystic exocrine structures. White scale bars = 100 μm. Red scale bars = 2 μm.

Through our analysis of tissue architecture in the *Cdh1*-deleted postnatal pancreas, the presence of both cystic acinar tissue and dilated ductal morphology was evident by P4 (Fig. 2F, Supp. Fig. 2D). To further investigate the basis for this dysregulation, we sought to examine whether issues with intercellular contacts were apparent at P0, and whether these changed over the course of neonatal development. While P0 control tissue displayed stereotypical exocrine morphology in which acinar cells were arranged into precisely organized, apically-constricted acini (Fig. 3A,A’), these structures were notably disrupted in the *Cdh1*^*ΔPan/ΔPan*^ pancreas tissue (Fig. 3B,B’). To determine whether these structural alterations were the result of improper AJ formation, we used transmission electron microscopy (TEM) to visualize the intercellular ultrastructures of adjoining exocrine cells. At P0, acinar and ductal cells in control tissues were characterized by close associations between cells, with the presence of tight junctions (TJs) and AJs located on their apicolateral membranes (Fig. 3E-E”). In contrast, the *Cdh1*^*ΔPan/ΔPan*^ pancreas exhibited dramatic dysregulation of this intracellular architecture at the ultrastructural level (Fig. 3F-F”), notably lacking in these junctional structures. Collectively, these results indicate that despite the normal gross morphology observed in *Cdh1*^*ΔPan/ΔPan*^ pancreata at the whole-organ level (Fig. 2B), the architecture of P0 exocrine tissue following *Cdh1* deletion is compromised at the ultrastructural level. Over time, this dysregulation gets more apparent, as evidenced by P4 histological (Fig. 3C,D) and ultrastructural (Fig. 3G,H) analyses.

In addition, we observed different levels of architectural dysregulation across pancreas lineages, with acinar-acinar contacts showing the highest levels of disordered architecture (Supp. Fig. 3A), and endocrine-endocrine contacts showing the lowest (Supp. Fig. 3C). These observations suggest lineage-specific requirements for E-cadherin in maintaining proper architecture between cells in the pancreas. This finding is also consistent with our observation that the exocrine pancreas is affected by *Cdh1* deletion to a greater degree than the endocrine pancreas.

While we observed a progressive breakdown in pancreatic architecture between P0 (Fig. 3I,J) and P4 (Fig. 3K,L) in *Cdh1*^*ΔPan/ΔPan*^ tissue, both the acinar and ductal compartments maintained their overall polarity, with the basal membrane marker Laminin localized to the basal membrane of Epcam-expressing acinar bundles. Collectively, these findings indicate that loss of E-cadherin during pancreas development results in the progressive dysregulation of postnatal exocrine architecture without affecting the apical-basal polarity of pancreas exocrine cells.

### E-cadherin deletion leads to abnormal activation of Wnt and YAP signaling in the postnatal pancreas

**Figure 4:**
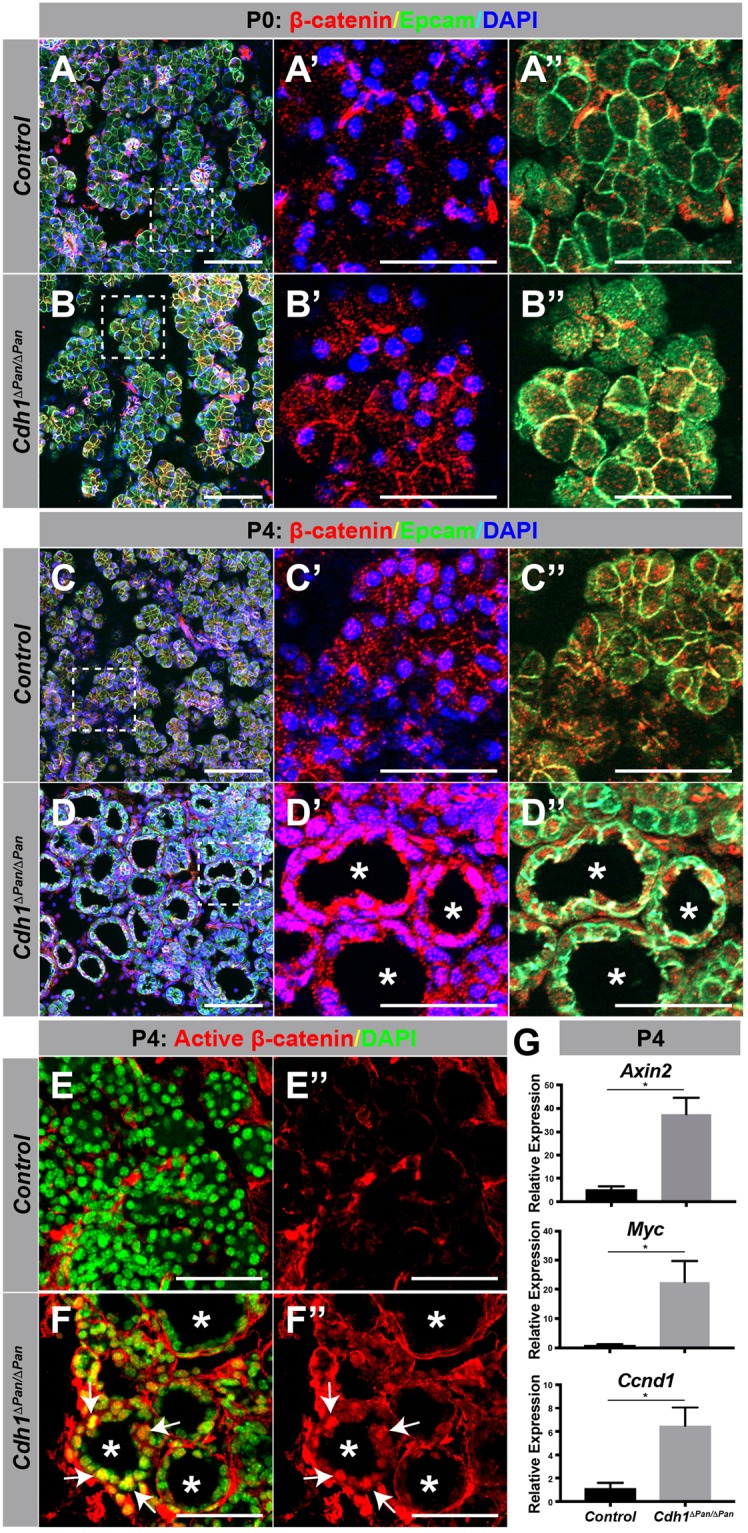
E-cadherin deletion leads to an increase of nuclear β-catenin localization in the P4 pancreas. (**A**–**D**) Immunofluorescence analysis of β-catenin (red), Epcam (green), and DAPI (blue) in the control (**A**,**C**) and *Cdh1*^*ΔPan/ΔPan*^ (**B**,**D**) pancreas at P0 and P4. Dashed white bounding boxes indicate dimensions of the projected area for the individual panels to the right (A’–B”). (**E**,**F**) Immunofluorescence analysis of active β-catenin (red), and DAPI (green) in the control (**E**) and *Cdh1*^*ΔPan/ΔPan*^ (**F**) pancreas at P4. White asterisks indicate dilated/cystic exocrine structures. White arrows indicate nuclear β-catenin. White scale bars = 100 μm. (**G**) qRT-PCR analysis of the Wnt targets *Axin2*, *Myc*, and *Ccnd1*. Histograms represent mean ± SEM of at least three independent determinations. For 2-tailed t-tests, *p < 0.05 compared to control.

The dramatic structural changes observed in *Cdh1*^*ΔPan/ΔPan*^ exocrine tissue prompted us to investigate whether exocrine homeostasis signaling was also affected. E-cadherin plays significant roles in the maintenance of epithelial homeostasis, both as a binding partner for cytoplasmic AJ components and as a regulator for intracellular contact inhibition. Through its role as an integral structural component of AJs, E-cadherin complexes with β-catenin, competitively inhibiting the ability of β-catenin to activate the canonical Wnt signaling pathway^13^. To assess whether *Cdh1* deletion resulted in abnormalities to β-catenin expression and Wnt signaling, we analyzed *Cdh1*^*ΔPan/ΔPan*^ and control tissues at P0 and P4. In the P0 pancreata, we observed the same β-catenin expression pattern exhibited across both *Cdh1*^*ΔPan/ΔPan*^ and control tissues (Fig. 4A,B). In P4 *Cdh1*^*ΔPan/ΔPan*^ pancreata however, subcellular localization of β-catenin appeared to differ from control tissue. In control pancreata, β-catenin expression was apparent in the vicinity of Epcam, suggesting primarily membrane localization (Fig. 4C). In contrast, *Cdh1*^*ΔPan/ΔPan*^ pancreata exhibited an increase in the amount of β-catenin appearing in both the cytosolic and nuclear compartment (Fig. 4D). To determine whether nuclear translocation of β-catenin was indeed occurring, we stained for the active, unphosphorylated form of β-catenin; and revealed a higher degree of nuclear localization for the *Cdh1*^*ΔPan/ΔPan*^ pancreas in comparison to control tissues (Fig. 4E,F). In addition, a significant upregulation of the Wnt pathway genes *Axin2*, *Myc*, *and Ccnd1* was observed in P4 *Cdh1*^*ΔPan/ΔPan*^ pancreata (Fig. 4G). Together, our data suggests that the postnatal *Cdh1*^*ΔPan/ΔPan*^ pancreas is characterized by activation of the Wnt signaling pathway.

**Figure 5:**
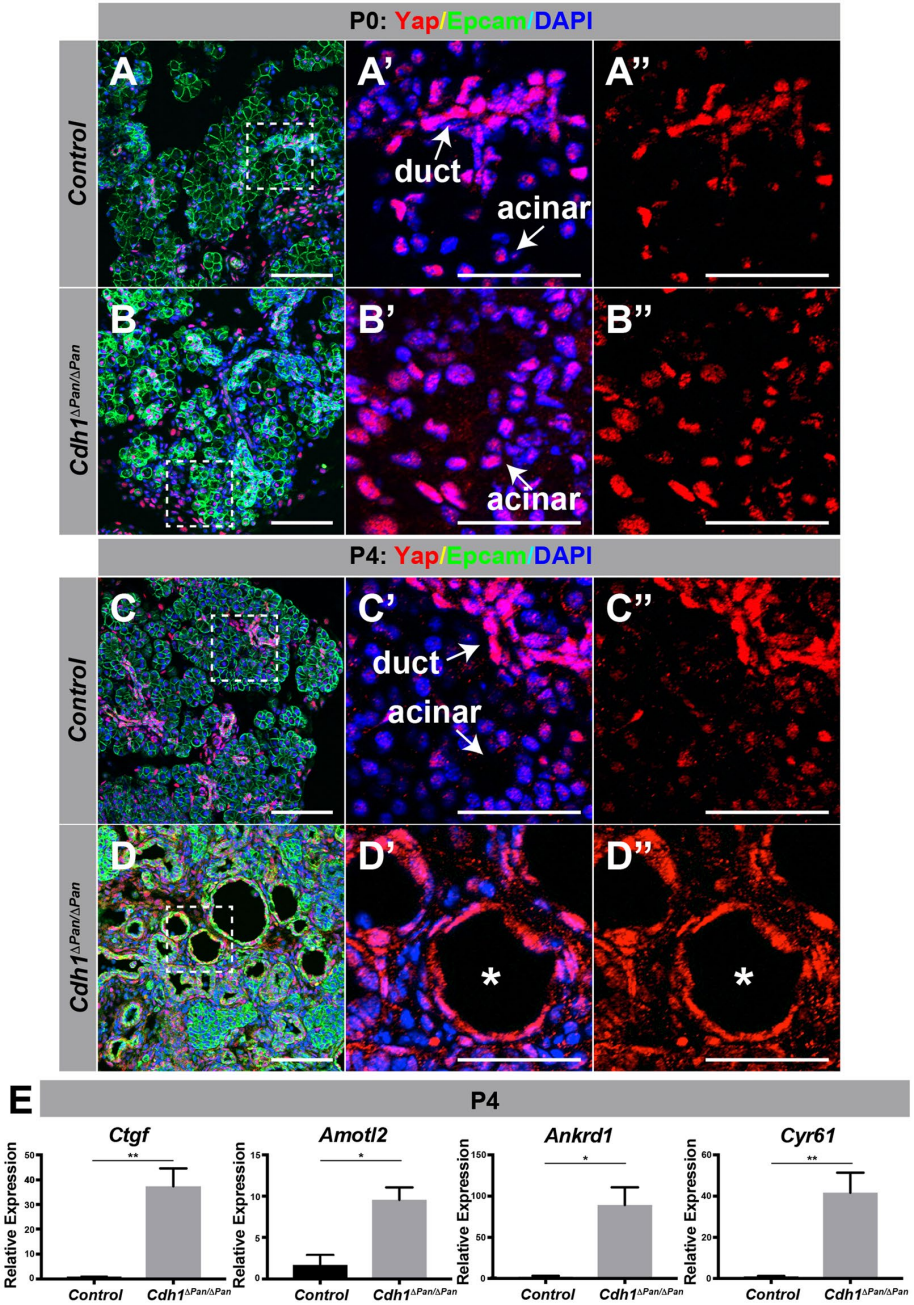
E-cadherin deletion promotes upregulated YAP signaling in the postnatal pancreas. (**A**–**D**) Immunofluorescence analysis of Yap (red), Epcam (green), and DAPI (blue) in the control (**A**,**C**) or *Cdh1*^*ΔPan/ΔPan*^ (**B**,**D**) pancreas at P0 and P4. Dashed white bounding boxes indicate dimensions of the projected area for each individual panel to the right (A’–D”). White scale bars = 100 μm. White asterisks indicate dilated/cystic exocrine structures. (**E**) qRT-PCR analysis of the YAP transcription effectors *Ctgf*, *Amotl2*, *Ankrd1* and *Cyr61*. Histograms represent mean ± SEM of at least three independent determinations. For 2-tailed t-tests, *p < 0.05, **p < 0.01, ***p < 0.001 compared to control.

In parallel to the Wnt signaling pathway, the Hippo/YAP signal transduction pathway is also strongly dependent on the establishment and maintenance of intracellular junctions. Consistent with this notion, we observed an increase in nuclear-localized Yap proteins in *Cdh1*^*ΔPan/ΔPan*^ acinar cells at both P0 and P4 (Fig. 5A–D). Further, significant increases in the expression of YAP signaling target genes *Ctgf*, *Amotl2*, *Ankrd1* and *Cyr61* were also observed at P4 (Fig. 5E). This result indicates that upon deletion of *Cdh1*, activation of YAP signaling occurs throughout postnatal development in the *Cdh1*^*ΔPan/ΔPan*^ exocrine pancreas. These findings are in agreement with the current understanding of the role which E-cadherin plays as an upstream regulator of Hippo/YAP signaling^20^. Collectively, these results indicate that both the Wnt and YAP signaling pathways are activated in the *Cdh1*^*ΔPan/ΔPan*^ pancreas.

### E-cadherin deletion promotes the development of acinar-to-ductal metaplasia (ADM) in the postnatal pancreas

**Figure 6:**
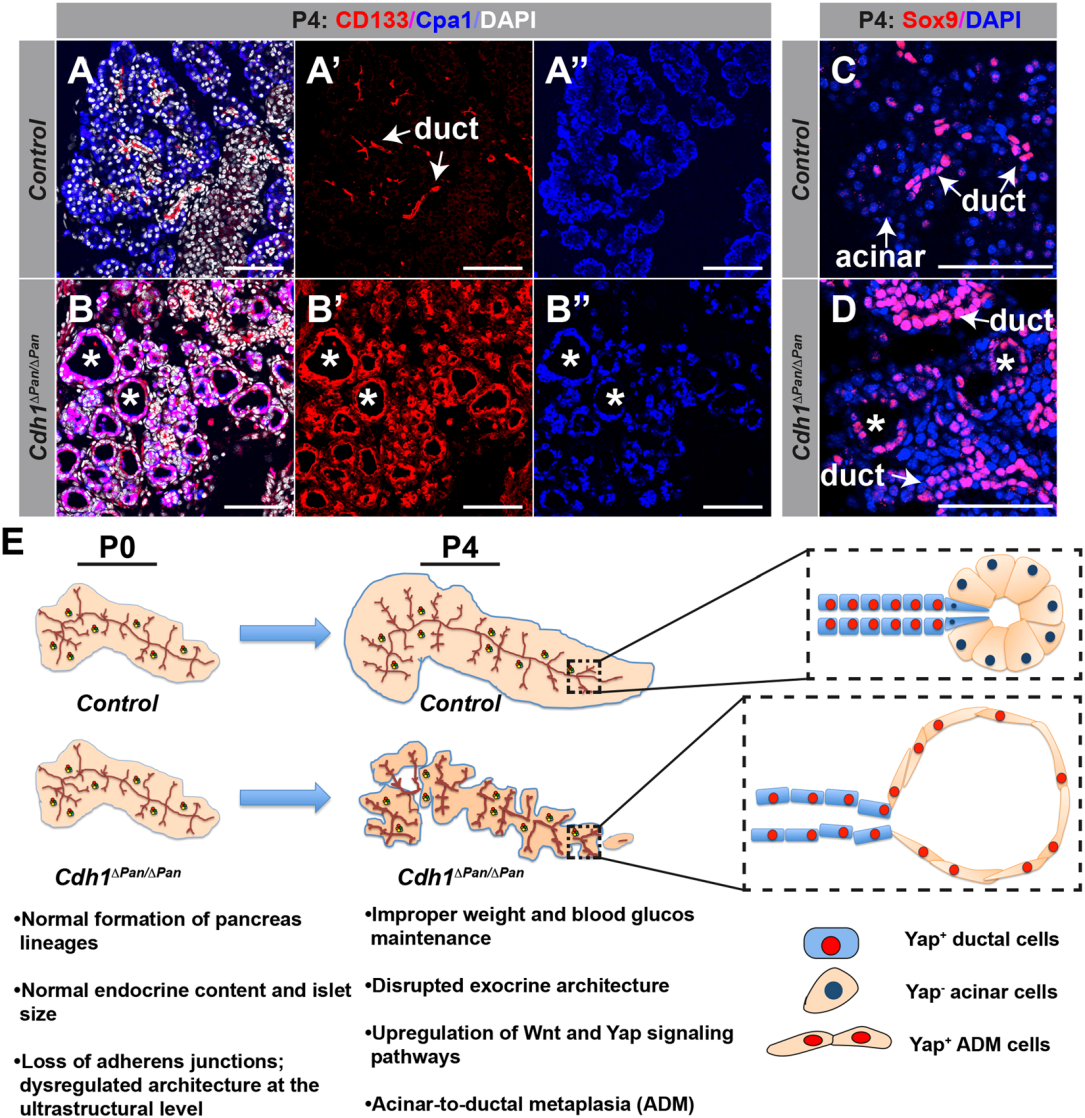
E-cadherin deletion promotes the development of acinar-to-ductal metaplasia in the postnatal pancreas. (**A**,**B**) Immunofluorescence analysis of ductal (CD133, red), acinar (Cpa1, blue), and nuclear (DAPI, white) markers in control (**A**) and *Cdh1*^*ΔPan/ΔPan*^ (**B**) pancreata at P4. White asterisks indicate dilated/cystic exocrine structures. (**C**,**D**) Immunofluorescence staining of ductal (Sox9, red) and nuclear (DAPI, blue) markers in control (**C**) and *Cdh1*^*ΔPan/ΔPan*^ (**D**) pancreata at P4. White arrows indicate pancreatic ductal or acinar cells, as indicated. White asterisks indicate dilated/cystic exocrine structures. White scale bars = 100 μm. (**E**) Schematic showing the progressive breakdown of the postnatal *Cdh1*^*ΔPan/ΔPan*^ pancreas architecture in comparison to controls.

Aberrant activation of the Wnt and Hippo/YAP pathways has been noted in a number of different pathogenic contexts, including the progression of chronic pancreatitis and metastatic pancreatic ductal adenocarcinoma (PDAC)^21–23^. Given the observed activation of these pathways in the *Cdh1*^*ΔPan/ΔPan*^ pancreas, we next sought to determine whether *Cdh1*^*ΔPan/ΔPan*^ exocrine tissue undergoes any degree of pathogenic change. During the onset of chronic pancreatitis and PDAC, a common pathological feature is ADM, in which pancreatic acinar cells adopt the characteristics of ductal cells. Normally, no colocalization of ductal (CD133) and acinar (Carboxypeptidase A1; Cpa1) markers is observed in control animals (Fig. 6A). In contrast, widespread colocalization of these markers in cystic acinar clusters was apparent throughout the exocrine pancreas in *Cdh1*^*ΔPan/ΔPan*^ tissue (Fig. 6B). In *Cdh1*^*ΔPan/ΔPan*^ pancreas tissue, we observed consistent evidence of ductal cells that contained large numbers of secretory granules, a feature typically associated with acinar cells. In contrast, control ductal cells contained virtually no secretory granules (Fig. S4A,B). In addition, we observed an upregulation of the duct-specific transcription factor Sox9 in cystic acinar clusters (Fig. 6C,D, asterisks). This finding supports the notion that ADM was occurring throughout the *Cdh1*^*ΔPan/ΔPan*^ exocrine tissue, as Sox9 is consistently upregulated during the de-/transdifferentiation of the cells^24–26^. Collectively, our results indicate that *Cdh1* deletion promotes ADM in the neonatal pancreas.

## Discussion

Traditionally, the adherens junction (AJ) structure has been considered critical to the maintenance of epithelial tissues throughout the body. At AJs, catenins bind to the cytoplasmic domain of E-cadherin, and link cell adhesion complexes to the intracellular actin cytoskeleton^27,28^. Aside from their roles as structural constituents, catenins have been implicated in complementary signaling roles related to growth and development. For example, deletion of either α-, β-, or δ- catenin results in abnormal pancreas development^17–19^. Here, we observed that E-cadherin deletion in the pancreas resulted in an absence of overt phenotypes at birth, followed by a dramatic breakdown in postnatal pancreas architecture over the ensuing four day period (Fig. 6E). This breakdown appeared to be entirely restricted to the exocrine pancreas, and though we cannot discount the possibility of alterations to endocrine functional capacity, we believe that the observed hypoglycemic phenotype in P4 *Cdh1*^*ΔPan/ΔPan*^ animals likely resulted from nutritional insufficiency, caused by an inability of the exocrine pancreas to function properly. Although ultrastructural defects were observed at P0 using TEM, large-scale defects in pancreas architecture were largely absent until a later postnatal period (P4). We propose that this change is likely correlated to the drastic increase in both organ growth and exocrine functional demands which occur after birth. Similar results have been observed in the developing mammary gland following E-cadherin deletion, in which dramatic collapse of exocrine architecture was initiated at the time of parturition, resulting in improper maintenance of the mammary epithelium^29^. While compensatory expression of other adhesion molecules may suffice to maintain epithelial function in the absence of E-cadherin to some extent, our results suggest that such alterations are functionally inadequate to support exocrine architecture. Indeed, our study consistently revealed a general increase in the expression of Epcam in the *Cdh1*^*ΔPan/ΔPan*^ postnatal pancreas (Figs 3–5), suggesting that compensatory expression may have in fact been occurring. This functional insufficiency of compensatory adhesion molecules has also been observed previously in mammary tissue, in which genetic replacement of E-cadherin with N-cadherin led to disruption of epithelial homeostasis^30^.

Another significant role of E-cadherin is to mediate cell contact inhibition, in which cells become immobilized following the formation of intercellular adhesions^31,32^. More recently, the role of AJs as regulators of contact inhibition has been addressed, with evidence suggesting that E-cadherin acts as a direct mediator of contact inhibition via Hippo signaling, and thus the regulation of subcellular Yap localization^14^. In the pancreas, Hippo signaling also plays a crucial role in controlling organ size and maintaining exocrine architecture^33^. Upon pancreas-specific deletion of the Hippo signaling mediators Mst1/2, George *et al*. showed that blocking Hippo activity results in tissue disorganization, characterized by an abundance of exocrine “transitional structures”, which demonstrated mixed acinar and ductal phenotypes, as well as patterns of pancreatitis-like features^33^. Similarly, we found that *Cdh1* deletion in the pancreas induced pancreatitis-like phenotypes, as well as an increase in the transcription of YAP targets. Thus, we speculate that the cystic phenotype observed in the *Cdh1*^*ΔPan/ΔPan*^ pancreas could be due to the disruption of the Hippo/YAP pathway.

Recently, it has been shown that crosstalk between the Hippo/YAP and Wnt signaling pathways regulates mammary gland homeostasis^34^. In the present study, we found that YAP signaling activity appeared to be overexpressed at P0, but the Wnt pathway was not shown to be activated at that time point, and instead became overexpressed later in postnatal development at P4 (Figs 4 and 5). To reconcile this asynchronous signaling activity, we propose that at P0, the YAP pathway may exert an inhibitory effect on Wnt signaling through the noncanonical Wnt pathway^34^. At this time point, YAP-induced noncanonical Wnt inhibition may be sufficient to override positive signaling through the canonical Wnt pathway, preventing aberrant activation of downstream Wnt targets. At P4, when both YAP and Wnt pathways are subsequently activated, the inhibitory regulation promoted by the YAP-induced noncanonical Wnt pathway may no longer be sufficient to disallow the activation of the canonical Wnt pathway. We reason that this would result in a situation in which both YAP- and Wnt-induced growth signaling pathways are promoted; though speculative, such an interplay is reasonable given our current understanding of the interdependence between the two pathways^34,35^. Whether these mechanisms also contribute to the phenotypes observed in *Cdh1*^*ΔPan/ΔPan*^ mutants is currently an open question.

Given its role in maintaining tissue homeostasis, the protective role of E-cadherin in suppressing cancer progression has been proposed^36–43^. Indeed, when combined with cancer-causing mutations, E-cadherin has been shown to play a significant protective role in maintaining tissue integrity across a range of organ systems, including the mammary gland, stomach, liver, skin, uterus, lung and nervous system^36–43^. Clinically, E-cadherin loss has been correlated with poor prognoses in pancreatic cancer patients^44^. However, whether loss of E-cadherin alone is sufficient to initiate pancreatic cancer remains a subject of inquiry. Our study suggests that E-cadherin deletion may predispose acinar cells to lose their terminal identity, and become more duct-like in nature, a process referred to as ADM. When ADM is combined with either aberrant growth factor signaling or oncogenic mutations, the occurrence of pancreatic cancer precursors such as pancreatic intraepithelial neoplasia (PanIn) is significantly increased^45,46^.

In the *Cdh1*^*ΔPan/ΔPan*^ pancreas, we observed that ADM occurs rapidly, within the span of 4 days postnatally. Given the known relationship between ADM and the progression of metastatic cancers, as well as the known tumor suppressor function of E-cadherin in other tissues, pathogenic E-cadherin inactivation may be sufficient to initiate pancreatic cancer. Future studies should aim to better characterize this hypothesis using both genetic and pharmacological approaches in which both E-cadherin and other tumor suppressors are inactivated in unison. In addition, the use of lineage-specific inducible deletion models (e.g. *Sox9-CreER* or *Ptf1a-CreER*) would also provide valuable insights into the role which E-cadherin plays in maintaining exocrine homeostasis in the mature adult pancreas. Such insights will be of great clinical utility, not only in further delineating the role which E-cadherin plays in tissue architecture and homeostasis, but also in informing future studies aimed at identifying improved prognostic biomarkers for PDAC, as well as therapeutic candidates for its treatment.

## Materials and Methods

### Mouse Strains

All animal experiments described herein were approved by the City of Hope Institutional Animal Care and Use Committees, and were performed in accordance with all relevant guidelines and regulations. Mice carrying *Pdx1-*Cre and *Ecad*^*flox/flox*^ alleles have been previously described^29,47^. Pancreas-specific *Cdh1*-deleted mouse strain (heretofore regarded as *Cdh1*^*ΔPan/ΔPan*^), mutant embryos were generated by mating *Pdx1-Cre;Cdh1*^*flox/+*^ males to *Ecad*^*flox/flox*^ females.

### Immunohistochemistry

Tissue was prepared and immunofluorescence staining performed as described previously^48^. Primary and secondary antibodies are listed in Supplementary Table 1. ApoTome images were captured on a Zeiss Axio-Observer-Z1 microscope with Zeiss Zen and figures prepared using Adobe Photoshop/Illustrator CC 2015. Pancreata of postnatal mice were dissected with stomach, duodenum and spleen for whole-mount light microscopy imaging. The stomach was removed prior to sectioning, and tissue was sectioned at 10 μm. For morphometric analyses, we used Image-Pro Premier v.9.2 to measure the surface area of specific areas. Four evenly spaced sections per condition and at least three animals per condition were analyzed for all experiments described.

### Whole-Mount Immunofluorescence Staining

Pancreas tissue was dissected and fixed overnight in 4% paraformaldehyde (PFA), dehydrated in a series of methanol washes and bleached in a Dent’s Bleach solution to remove background fluorescence^49^. Samples were then rehydrated using an inverse series of methanol washes, and stained with primary antibodies for Mucin1 and Epcam overnight. Samples were then washed in a 0.15% tween-PBS solution and stained with secondary antibodies overnight. Following an additional fixation step, samples were exposed to BABB clearing solution (two parts benzyl benzoate and one part benzyl alcohol) and imaged using a Zeiss LSM880 confocal microscope.

### Transmission Electron Microscope Imaging

Pancreas tissue was dissected from P0 and P4 animals into fixative (2% glutaraldehyte in 0.1 M Cacodylate buffer (Na(CH_3_)_2_AsO_2_·3H_2_O), pH7.2), cut into ~1 mm^3^ sections, and placed at 4 °C overnight. Tissue was washed 3x with 0.1 M Cacodylate buffer, post-fixed with OsO_4_ in 0.1 M Cacodylate buffer for 30 min and washed three times with 0.1 M Cacodylate buffer. The samples were then dehydrated through 60%, 70%, 80%, 95% ethanol, 100% absolute ethanol (twice), propylene oxide (twice), and were left in propylene oxide/Eponate (1:1) overnight at room temperature in sealed vials. The next day the vials were left open for 2–3 hours to evaporate the propylene oxide. The samples were infiltrated with 100% Eponate and polymerized at ~64 °C for 48 hours. Ultra-thin sections (~70 nm thick) were cut using a Leica Ultra cut UCT ultramicrotome with a diamond knife, picked up on 200 mesh copper EM grids. Grids were stained with 2% uranyl acetate for 10 minutes followed Reynold’s lead citrate staining for 1 minute. Electron microscopy was done on an FEI Tecnai 12 transmission electron microscope equipped with a Gatan Ultrascan 2 K CCD camera.

### qRT-PCR and Gene Expression Analysis

Total RNA was isolated from dissected mouse pancreata into an RLT buffer containing 1% β-mercaptoethanol, and purified using a PureLink™ RNA Micro Kit (Invitrogen, Carlbad, CA). For qRT-PCR analysis, cDNA was synthesized using Superscript III First Strand cDNA Kit (Invitrogen) for each individual sample. qRT-PCR was performed using four replicates for each condition, using SYBR Green according to the manufacturer’s specifications (Applied Biosystems). Relative quantification of qRT-PCR was normalized using Pfaffl methods^50^.

### Blood Glucose Measurements

All blood glucose measurements were performed using a Freestyle Lite blood glucose meter (Abbott Laboratories, Chicago, IL). Blood glucose was taken immediately before animals were sacrificed for immunohistochemical analysis, with no prior fasting period.

### Statistical Analysis

Statistical significance of all measurements was assessed using a one-way analysis of variance (ANOVA) followed by a student’s *t*-test to compare control and treatment groups, and represents a minimum of at least three independent measurements. All values are represented as mean ± SEM. For 2-tailed t-tests, *p < 0.05, **p < 0.01, ***p < 0.001 compared to control.

## Electronic supplementary material

Supplementary information

## References

[1] Acloque H, Adams MS, Fishwick K, Bronner-Fraser M, Nieto MA (2009). Epithelial-mesenchymal transitions: the importance of changing cell state in development and disease. J Clin Invest.

[2] Andrew DJ, Ewald AJ (2010). Morphogenesis of epithelial tubes: Insights into tube formation, elongation, and elaboration. Dev Biol.

[3] Guillot C, Lecuit T (2013). Mechanics of epithelial tissue homeostasis and morphogenesis. Science.

[4] Macara IG, Guyer R, Richardson G, Huo Y, Ahmed SM (2014). Epithelial homeostasis. Curr Biol.

[5] Wang CC, Jamal L, Janes KA (2012). Normal morphogenesis of epithelial tissues and progression of epithelial tumors. Wiley Interdiscip Rev Syst Biol Med.

[6] Villasenor A, Chong DC, Henkemeyer M, Cleaver O (2010). Epithelial dynamics of pancreatic branching morphogenesis. Development.

[7] Halbleib JM, Nelson WJ (2006). Cadherins in development: cell adhesion, sorting, and tissue morphogenesis. Genes Dev.

[8] Lewis JE, Jensen PJ, Johnson KR, Wheelock MJ (1994). E-cadherin mediates adherens junction organization through protein kinase C. J Cell Sci.

[9] Takeichi M (2014). Dynamic contacts: rearranging adherens junctions to drive epithelial remodelling. Nat Rev Mol Cell Biol.

[10] Hartsock A, Nelson WJ (2008). Adherens and tight junctions: structure, function and connections to the actin cytoskeleton. Biochim Biophys Acta.

[11] Stepniak E, Radice GL, Vasioukhin V (2009). Adhesive and signaling functions of cadherins and catenins in vertebrate development. Cold Spring Harb Perspect Biol.

[12] van Roy F (2014). Beyond E-cadherin: roles of other cadherin superfamily members in cancer. Nat Rev Cancer.

[13] Heuberger J, Birchmeier W (2010). Interplay of cadherin-mediated cell adhesion and canonical Wnt signaling. Cold Spring Harb Perspect Biol.

[14] Kim NG, Koh E, Chen X, Gumbiner BM (2011). E-cadherin mediates contact inhibition of proliferation through Hippo signaling-pathway components. Proc Natl Acad Sci USA.

[15] Baumgartner BK, Cash G, Hansen H, Ostler S, Murtaugh LC (2014). Distinct requirements for beta-catenin in pancreatic epithelial growth and patterning. Dev Biol.

[16] Murtaugh LC (2008). The what, where, when and how of Wnt/beta-catenin signaling in pancreas development. Organogenesis.

[17] Murtaugh LC, Law AC, Dor Y, Melton DA (2005). Beta-catenin is essential for pancreatic acinar but not islet development. Development.

[18] Jimenez-Caliani AJ (2017). alphaE-Catenin Is a Positive Regulator of Pancreatic Islet Cell Lineage Differentiation. Cell Rep.

[19] Hendley AM (2015). p120 Catenin is required for normal tubulogenesis but not epithelial integrity in developing mouse pancreas. Dev Biol.

[20] Gumbiner BM, Kim NG (2014). The Hippo-YAP signaling pathway and contact inhibition of growth. J Cell Sci.

[21] Morvaridi S, Dhall D, Greene MI, Pandol SJ, Wang Q (2015). Role of YAP and TAZ in pancreatic ductal adenocarcinoma and in stellate cells associated with cancer and chronic pancreatitis. Sci Rep.

[22] Zhan T, Rindtorff N, Boutros M (2017). Wnt signaling in cancer. Oncogene.

[23] Zhang W (2014). Downstream of mutant KRAS, the transcription regulator YAP is essential for neoplastic progression to pancreatic ductal adenocarcinoma. Sci Signal.

[24] Kopp JL (2012). Identification of Sox9-dependent acinar-to-ductal reprogramming as the principal mechanism for initiation of pancreatic ductal adenocarcinoma. Cancer Cell.

[25] Pinho AV, Chantrill L, Rooman I (2014). Chronic pancreatitis: a path to pancreatic cancer. Cancer Lett.

[26] Pinho AV (2011). Adult pancreatic acinar cells dedifferentiate to an embryonic progenitor phenotype with concomitant activation of a senescence programme that is present in chronic pancreatitis. Gut.

[27] Gumbiner BM (2000). Regulation of cadherin adhesive activity. J Cell Biol.

[28] Jamora C, Fuchs E (2002). Intercellular adhesion, signalling and the cytoskeleton. Nat Cell Biol.

[29] Boussadia O, Kutsch S, Hierholzer A, Delmas V, Kemler R (2002). E-cadherin is a survival factor for the lactating mouse mammary gland. Mech Dev.

[30] Kotb AM, Hierholzer A, Kemler R (2011). Replacement of E-cadherin by N-cadherin in the mammary gland leads to fibrocystic changes and tumor formation. Breast Cancer Res.

[31] Fagotto F, Gumbiner BM (1996). Cell contact-dependent signaling. Dev Biol.

[32] Huttenlocher A (1998). Integrin and cadherin synergy regulates contact inhibition of migration and motile activity. J Cell Biol.

[33] George NM, Day CE, Boerner BP, Johnson RL, Sarvetnick NE (2012). Hippo signaling regulates pancreas development through inactivation of Yap. Mol Cell Biol.

[34] Park HW (2015). Alternative Wnt Signaling Activates YAP/TAZ. Cell.

[35] Attisano L, Wrana JL (2013). Signal integration in TGF-beta, WNT, and Hippo pathways. F1000Prime Rep.

[36] Shimada S (2012). Synergistic tumour suppressor activity of E-cadherin and p53 in a conditional mouse model for metastatic diffuse-type gastric cancer. Gut.

[37] Ceteci F (2007). Disruption of tumor cell adhesion promotes angiogenic switch and progression to micrometastasis in RAF-driven murine lung cancer. Cancer Cell.

[38] Derksen PW (2011). Mammary-specific inactivation of E-cadherin and p53 impairs functional gland development and leads to pleomorphic invasive lobular carcinoma in mice. Dis Model Mech.

[39] Derksen PW (2006). Somatic inactivation of E-cadherin and p53 in mice leads to metastatic lobular mammary carcinoma through induction of anoikis resistance and angiogenesis. Cancer Cell.

[40] Nakagawa H (2014). Loss of liver E-cadherin induces sclerosing cholangitis and promotes carcinogenesis. Proc Natl Acad Sci USA.

[41] Schneider MR (2014). Evidence for a role of E-cadherin in suppressing liver carcinogenesis in mice and men. Carcinogenesis.

[42] Stodden GR (2015). Loss of Cdh1 and Trp53 in the uterus induces chronic inflammation with modification of tumor microenvironment. Oncogene.

[43] Xing X (2013). The prognostic value of E-cadherin in gastric cancer: a meta-analysis. Int J Cancer.

[44] Winter JM (2008). Absence of E-cadherin expression distinguishes noncohesive from cohesive pancreatic cancer. Clin Cancer Res.

[45] Navas C (2012). EGF receptor signaling is essential for k-ras oncogene-driven pancreatic ductal adenocarcinoma. Cancer Cell.

[46] Storz P (2017). Acinar cell plasticity and development of pancreatic ductal adenocarcinoma. Nat Rev Gastroenterol Hepatol.

[47] Hingorani SR (2003). Preinvasive and invasive ductal pancreatic cancer and its early detection in the mouse. Cancer Cell.

[48] Shih HP, Gross MK, Kioussi C (2007). Expression pattern of the homeodomain transcription factor Pitx2 during muscle development. Gene Expr Patterns.

[49] Becker K, Jahrling N, Kramer ER, Schnorrer F, Dodt HU (2008). Ultramicroscopy: 3D reconstruction of large microscopical specimens. J Biophotonics.

[50] Pfaffl MW (2001). A new mathematical model for relative quantification in real-time RT-PCR. Nucleic Acids Res.

