# Supplementary material for: Pancreatic Exocrine Tissue Architecture and Integrity are Maintained by E-cadherin During Postnatal Development
Source: Sci Rep. 2018 Sep 7;8:13451. doi: 10.1038/s41598-018-31603-2 (PMC6128895; doi:10.1038/s41598-018-31603-2)

Pancreatic Exocrine Tissue Architecture and Integrity are Maintained by E-cadherin During Postnatal Development

Jeffrey D. Serrill, Maike Sander, Hung Ping Shih

Supplementary Information

1. **Supplementary Table 1**

Antigen	Species	Source	Dilution
Active β-catenin	Mouse	Millipore	1:200
Amylase	Mouse	Santa Cruz	1:500
β-catenin	Rabbit	Cell Signaling	1:300
Chromogranin A	Goat	Santa Cruz	1:500
Cdh1	Rat	Cell Signaling	1:500
Cdh1	Goat	R&D	1:500
Cpa1	Goat	R&D	1:500
CD133	Rabbit	Abcam	1:200
YAP	Rabbit	Cell Signaling	1:300
Epcam	Rat	DSHB	1:100
Glucagon	Rabbit	Invitrogen	1:500
Insulin	Guinea Pig	Dako	1:500
Laminin 1	Rabbit	Sigma	1:1000
Mucin 1	Rabbit	Abcam	1:500
Nr5a2	Mouse	R&D	1:300
Pancreatic Polypeptide	Goat	Novus	1:500
Somatostatin	Goat	Santa Cruz	1:200
Sox9	Rabbit	Millipore	1:200
Trypsin	Sheep	R&D	1:300
Bio-DBA		Vector Laboratories	1:500

2. Supplementary Figure Legends

Supp. Figure 1. *Cdh1$^{\Delta Pan/\Delta Pan}$* mice exhibit normal endocrine development. Immunofluorescence analysis of Cdh1 (white) in the control (A) and *Cdh1$^{\Delta Pan/\Delta Pan}$* (B) pancreas at E11.5. Immunofluorescence analysis of acinar (amylase, green), ductal (DBA, white), and endocrine (chromogranin A, red) compartments of control (C) and *Cdh1$^{\Delta Pan/\Delta Pan}$* (D) pancreata at E15.5. Quantification of acinar (E) and endocrine (F) areas in control and *Cdh1$^{\Delta Pan/\Delta Pan}$* mice at E15.5. (G) Relative pancreas size in control and *Cdh1$^{\Delta Pan/\Delta Pan}$* mice at E15.5. (H-K) Immunofluorescence analysis of the endocrine markers insulin, glucagon, somatostatin, and pancreatic polypeptide (green) and Chromogranin A (red) in control (H,J) and *Cdh1$^{\Delta Pan/\Delta Pan}$* (I,K) pancreata at P0 and P4. Histograms represent mean ± SEM of at least three independent determinations. For 2-tailed t-tests, * = $p<0.05$, ** = $p<0.01$, *** = $p<0.001$ compared to control. White scale bars = 100 µm.

Supp. Figure 2. *Cdh1$^{\Delta Pan/\Delta Pan}$* mice exhibit normal expression patterns of acinar markers at P0. (A-F) Immunofluorescence analysis of the acinar markers Cpa1, Trypsin, and Nr5a2 (green), the epithelia cell marker Epcam (red) and nuclei (white, DAPI) in control (A,C,E) and *Cdh1$^{\Delta Pan/\Delta Pan}$* (B,D,F) mice at P0. (G,H) Whole-mount immunofluorescence images showing the distribution of ductal (Muc1, red) and exocrine (Epcam, green) compartments in the control (G) or *Cdh1$^{\Delta Pan/\Delta Pan}$* (H) pancreas at P0. White scale bars = 100 µm.

Supp. Figure 3. Exocrine tissues in *Cdh1$^{\Delta Pan/\Delta Pan}$* pancreata exhibit ultrastructural impairments at P0. Transmission electron micrographs of acinar (A), ductal (B), and endocrine (C) tissue in the P0 *Cdh1$^{\Delta Pan/\Delta Pan}$* pancreas. Higher magnification views of red and yellow inset boxes are shown to the right in (A'-C') and (A''-C''), respectively. Red scale bars = 2 µm.

Supp. Figure 4. E-cadherin deletion leads to acinar-to-ductal metaplasia (ADM) in the P4 pancreas. Transmission electron micrographs of representative

ductal structures in control (A) and *Cdh1*$^{\Delta Pan/\Delta Pan}$ (B) pancreata at P4. Dashed red boxes are projected in panels to the right (A',B'). Red scale bars = 2 µm.

3. Supplemetary Figures

Suppl. Figure 1

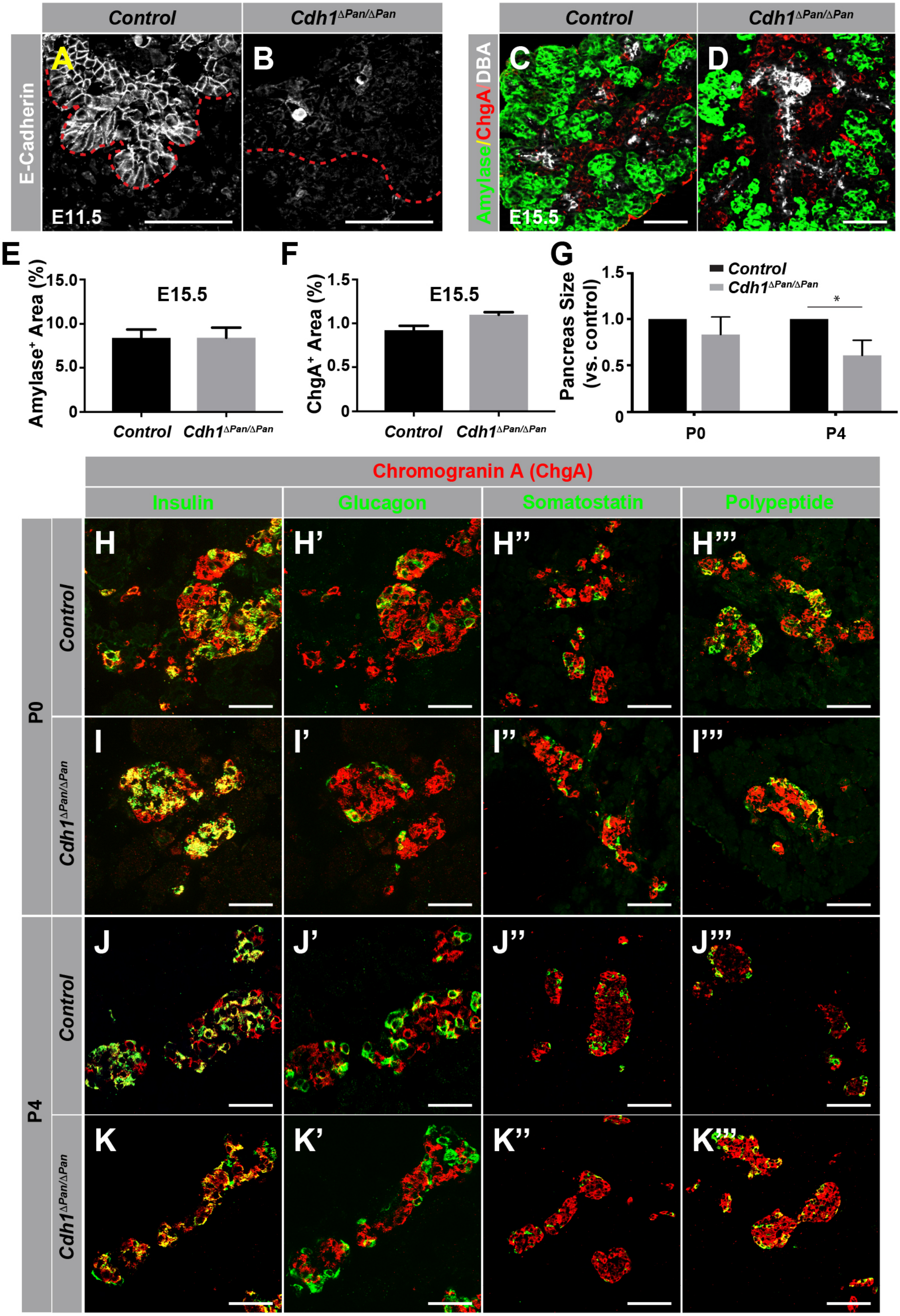

Suppl. Figure 2

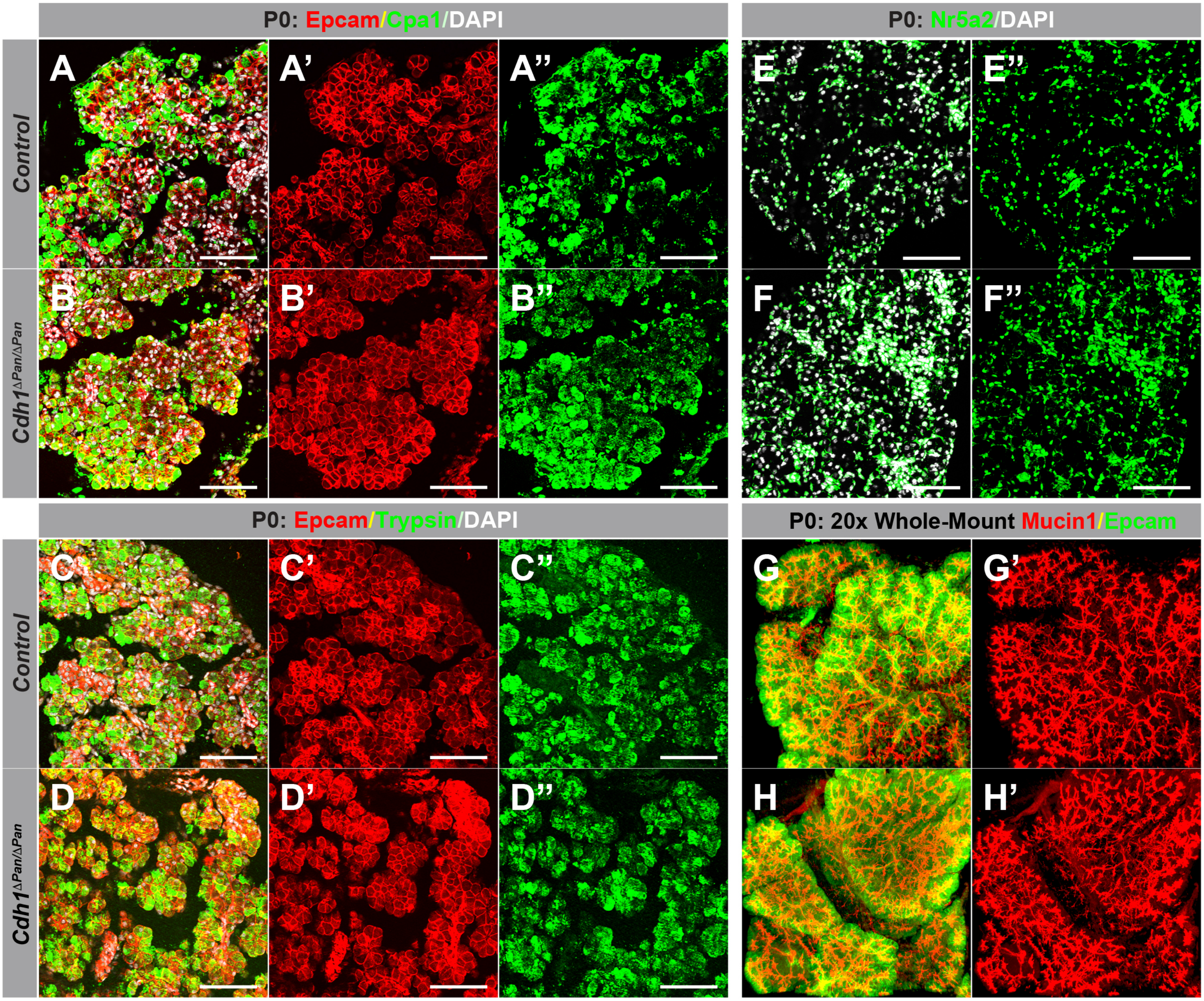

Suppl. Figure 3

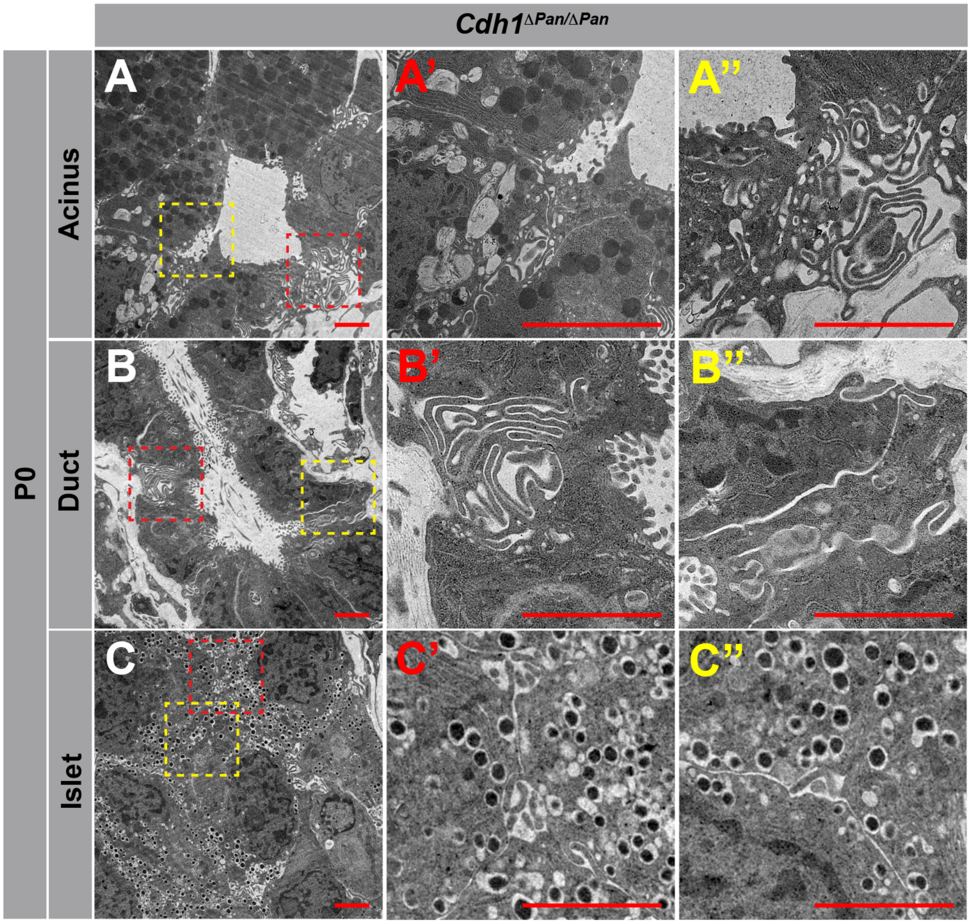

Suppl. Figure 4

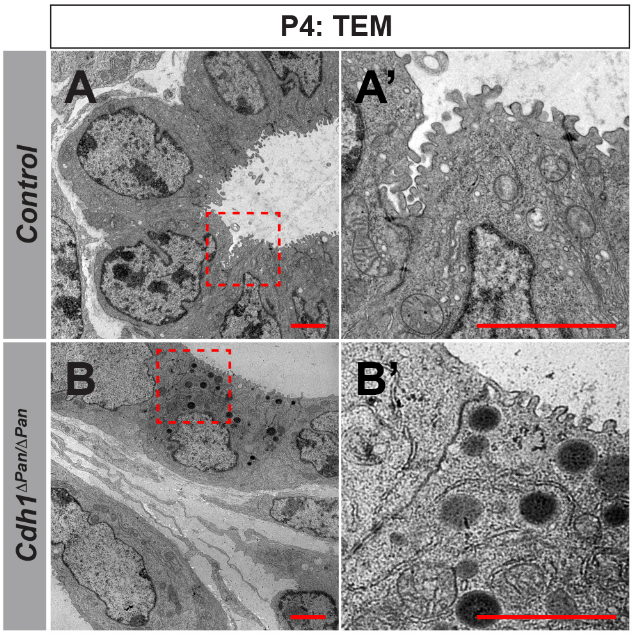

Supplement: Supplementary file 1 — Supplementary information [file 41598_2018_31603_MOESM1_ESM.pdf]
